# The impact of agricultural production diversity on farmer household dietary diversity: a case study of Nanjing City

**DOI:** 10.3389/fnut.2025.1493371

**Published:** 2025-04-30

**Authors:** Qi Shen, Tingting Qiang, Taiyang Zhong

**Affiliations:** School of Geography and Ocean Science, Nanjing University, Nanjing, China

**Keywords:** agricultural production diversity, dietary diversity, feasible generalized least squares method, simultaneous equation model, Nanjing

## Abstract

**Background/Objectives:**

China is one of the first developing countries to achieve poverty reduction goals, but there are still problems with unbalanced dietary structures in rural areas. The food consumption problem of rural residents is a major social issue that deserves attention, and food security should be guaranteed.

**Methods:**

This paper takes rural households in Nanjing as the research object and uses feasible generalized least squares and simultaneous equation models to explore the relationship between the diversity of rural household production and dietary diversity in Nanjing and analyze the differences in the impact of production diversity and market purchases on dietary diversity and other related influencing factors.

**Results:**

The results show that agricultural production has changed from “small and comprehensive” with diversity to relatively single “specialization”. The higher the diversity of crop production of rural households, the higher their dietary diversity. The dietary diversity of rural households in Nanjing is relatively low, and their food consumption structure is unreasonable.

**Conclusion:**

The impact of production diversity and market purchases on dietary diversity is different, and the positive impact of production diversity on dietary diversity is greater than that of market purchases. Encouraging farmers to apply for agricultural product certification, cultivating new types of professional farmers, and expanding channels for selling agricultural products can effectively improve the dietary diversity of rural households.

## 1 Introduction

China was the first developing country in the world to achieve the goal of reducing poverty, basically solving the problem of “having enough to eat,” and significantly improving the problem of nutritional deficiencies, such as low body weight and thinness, among people living in rural areas. Rural residents’ demand for food has changed from “enough to eat” to “enough to eat well.” This change reflects that rural residents are no longer satisfied with food security alone, but are more inclined to choose to consume more diversified food with higher nutritional value, such as increasing the consumption of food of animal origin (1). However, the Report on Nutrition and Chronic Diseases in China (2020) highlights that the unreasonable dietary structure of Chinese residents is a prominent problem, especially in rural areas (2). Unreasonable food consumption and dietary structures can lead to increased weight, obesity, diet-related chronic diseases, and other health issues, which are especially severe in rural areas. This exacerbates the health burden on rural residents, who often have limited access to medical and health resources compared to urban populations. Consequently, improving food consumption patterns and ensuring food security for rural residents is a major social issue in China. Attention must be focused on enhancing dietary practices in rural areas to safeguard the health of rural populations.

Household dietary diversity is recognized as a key factor influencing the quality of household diets and food security (3). According to the Food and Agriculture Organization (FAO), food security is defined when all people, at all times, have physical and economic access to sufficient, safe, and nutritious food that meets their dietary needs and food preferences for an active and healthy life (4). Thus, household dietary diversity is an important expression of household food security. Dietary diversity reflects access to a variety of foods and is a major dimension of food security. Therefore, it is crucial to conduct an in-depth analysis of the factors influencing household dietary diversity in rural areas of China.

In recent years, the impact of agricultural production diversity and other factors on dietary diversity has received extensive academic attention. In terms of influencing factors, scholars mainly focus on agricultural production diversity (5), market access (6), and agricultural commercialization (7), as well as household and individual socio-economic characteristics such as gender (8), age (9), years of education (10), household size (9), and household income (11). Some scholars assert that agricultural production diversity is a significant factor influencing dietary diversity (12). In contrast, others argue that market factors play a more substantial role in dietary diversity than agricultural production diversity (13), or that there is no correlation between the two (14). This suggests that the impact of agricultural production diversification on dietary diversity is complex and that the association between the two and the underlying causal mechanisms are unclear. Additionally, there is considerable debate over whether agricultural production diversity or market factors are more effective in enhancing dietary diversity. Some studies have significantly expanded the scope of research on agricultural production diversity and dietary diversity in China, extending it to a national level and focusing on the impact of agricultural production diversity on the consumption of animal-based foods among farmers (15). However, empirical studies examining the relationship between production diversity and dietary diversity based on micro-level data regarding individual farming households remain relatively scarce. Additionally, there is a lack of data addressing the status of Chinese farmers over the past 2 years.

Considering that peasants are not all pure subsistence farmers (16) and that they may engage in market transactions for buying or selling food, the relationship between agricultural production diversity and dietary diversity becomes more intricate. Existing research on how agricultural production diversity affects dietary diversity has produced inconsistent conclusions. Current views can be roughly categorized into four groups: The first view posits that production diversity and livestock ownership are the most important factors influencing dietary diversity (17). Studies suggest that agricultural production diversity has a significant positive impact on the dietary status of rural households (5, 9, 12, 18). Agricultural production diversity may influence dietary diversity through various pathways, such as directly promoting personal consumption and indirectly through income effects, reducing food prices, increasing the availability of nutritious agricultural products, enhancing food consumption, and increasing women’s control over community and household resources, knowledge, and status (5, 19). The second view argues that production diversity has an insignificant impact on dietary status (13, 14). Improvements in diet quality are attributed to increased income from cash crop sales rather than diversified subsistence production. In some cases, increasing agricultural production diversity is not the most effective way to improve household dietary diversity among small-scale farmers (13). The third view suggests that agricultural production diversity is less relevant to rural household dietary status, and that a substantial increase in the number of species produced is needed to produce meaningful changes in dietary diversity (20). The fourth view contends that production diversity is not the key factor affecting dietary diversity. Instead, other factors, such as economic growth (21–23), urbanization (24), market access (5, 13), roads, and other infrastructure (18) have significant impacts on dietary diversity. This view emphasizes that these factors, rather than production diversity, have a greater influence on dietary diversity. In studies of multiple regions of Indonesia, productive diversity, as measured by a simple species count, was positively correlated with most dietary indicators. When production diversity is measured in terms of the number of food groups produced, this association becomes insignificant in many cases (14). In South Africa, a positive correlation has been observed between production diversity and the dietary diversity of subsistence farmers, although dietary diversity also varies according to household demographics and socio-economic characteristics (25). Similarly, studies in India indicate that households with diverse agricultural production exhibit higher dietary diversity, with markets playing only an auxiliary role that is less significant than their own production (26). Overall, in developing countries, production diversity remains the primary factor influencing dietary diversity, while increased market participation acts as a secondary factor. China is a large country in terms of population and economy among developing countries, and it is worth considering whether market participation is the main influence factor of dietary diversity beyond production diversity. Therefore, the link between production diversity and dietary diversity is complex and there is still room for further research to explore.

Existing studies have discussed the impact of agricultural production diversity on farmers’ dietary diversity, but there are three main research gaps. Firstly, methodological and data challenges. Some research methods are one-sided and fail to adequately address heteroscedasticity and endogeneity caused by mutual causation in the data. Data collection challenges, including incompleteness and inaccuracy, affect the credibility and persuasiveness of the findings. Secondly, the limitation of concept definition. Most studies focus solely on a narrow agriculture concept, such as the planting industry or animal husbandry, without considering the broader scope of agriculture, which is insufficient to represent agricultural production in the relevant study areas. Thirdly, the lack of exploration into the specific mechanisms of action. While some studies indicate a positive correlation between agricultural diversity and dietary diversity among farmers, there is insufficient in-depth analysis of the micro-mechanisms underlying this relationship. A consensus on the core factors influencing dietary diversity among farming households has yet to be reached.

The production and management model, with the family as the basic unit, is still the mainstream agricultural production and management model in China today. In addition to relying on agricultural production, the dietary diversity of farmer households has gradually become dependent on agricultural markets. Especially in the context of China’s shift to specialized production and continuous commercialization of agriculture, the diversity of agricultural production and participation in the agricultural market will inevitably affect the dietary diversity of farmer households to different degrees, directly or indirectly, through different pathways, which in turn affects the dietary health and food security of people living in rural areas. Therefore, to better protect the dietary health and food security of the population, it is necessary to systematically assess the direction, degree, path and characteristics of the impact of agricultural production diversity and related influencing factors on the dietary diversity of farmer households. This assessment should be based on a sample of farmer households in Nanjing City to provide a realistic basis and decision-making reference for achieving sustainable food consumption and the Healthy China strategy.

Based on the above discussion, this paper attempts to analyze the relationship between agricultural production diversity and household dietary diversity by means of feasible generalized least squares (FGLS), and to deal with the possible endogeneity between the two using a simultaneous equation model. The possible contributions of this paper are mainly reflected in the following three aspects: firstly, this paper focuses on agricultural production diversity in the broad sense of agriculture, theoretically analyses and empirically tests the relationship between agricultural production diversity and dietary diversity in the broad sense of agriculture, and emphasizes forestry and fishery, which have been seldom researched in the past, thus expanding the scope of existing research on agricultural production diversity. Secondly, this paper eliminates the bias of heteroscedasticity and endogeneity by adopting feasible generalized least squares and a simultaneous equation model, enabling a more accurate measurement of the impact of agricultural production diversity on the dietary diversity of farmer households in rural areas of Nanjing. Finally, this paper constructs a conceptual framework of the relationship between agricultural production diversity as a production strategy and dietary diversity, incorporating market participation as a core factor rather than merely a control variable, distinguishing it from previous studies (27). This paper systematically analyzes the impact of agricultural production diversity on dietary diversity from two impact pathways: direct food consumption impact and indirect impact of commercialization for income. This approach more comprehensively distinguishes between self-sufficiency and agricultural commercialization as two distinct channels. Additionally, this study thoroughly investigates and considers the individual characteristics of each farmer, such as whether they are classified as new-type professional farmers, and the various inputs involved in the production of different agricultural products. This approach addresses the shortcomings of previous research, which often provided a generalized discussion of farmers as a collective group (15).

## 2 Literature review

**Figure 1:**
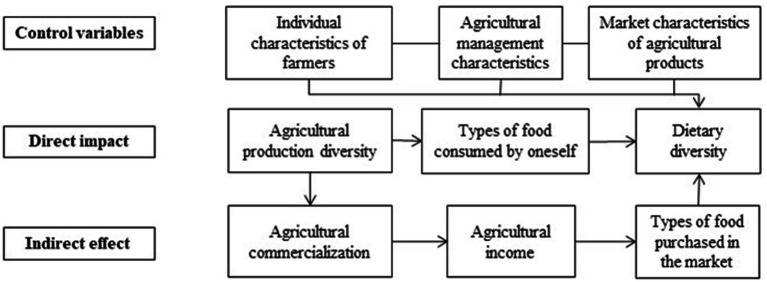
Pathways of agricultural production diversity on dietary diversity.

Relevant studies have shown that agricultural production by farm households has a crucial role to play when exploring the impacts on household dietary diversity. This study divides the effect of agricultural production diversity on dietary diversity into two pathways: direct food consumption impacts and indirect impacts of commercialization for income. Figure 1 illustrates how agricultural production diversity affects dietary diversity.

### 2.1 Direct impact

Agriculture as a direct source of food consumption. Firstly, agriculture produces agricultural products for food consumption, directly impacting household diets. The primary mode of agricultural production in China has long been small-scale farm management (28). Small-scale farmers are more likely to engage in diverse production with self-sufficiency characteristics, where the primary role of their agricultural activities is to meet their own food consumption needs (29). However, with the accelerated transfer of land use rights, as agricultural production gradually moves towards scale, mechanization, intensification, and commercialization, the importance of agricultural production activities has also evolved. Differences in agricultural production among farmers in various industries have become evident (30). Farmers in different agricultural sectors exhibit distinct food consumption patterns. For instance, farmers engaged in planting may have more diverse food sources and higher intake of nutrients such as vitamins and minerals, leading to a more balanced diet; farmers engaged in fishing may have increased consumption of aquatic products, with their intake of protein and other nutrients potentially exceeding that of farmers in other industries; similarly, farmers engaged in animal husbandry may consume more food of animal origin compared to those in other industries (31). Therefore, when food produced from agriculture is directly used for household consumption, it has a direct impact on the dietary diversity of the household. According to the survey, farming families mostly adopt crop rotation, with the types of agricultural products changing with the seasons. This practice not only enhances the diversity of agricultural production but also enables farming families to achieve higher dietary diversity and obtain a wider variety of nutrients to meet human needs.

### 2.2 Indirect effect

Indirect effects of agricultural commercialization in exchange for income. Market food purchases are an indispensable aspect of residents’ daily consumption behavior. Bennett’s law indicates that with rising incomes, residents will increasingly choose diverse and high-quality foods. As income rises, the consumption of staples such as rice and noodles decreases, while the intake of high nutritional value foods such as livestock and poultry products, fruits, dairy products, and aquatic products increases (66). Chinese residents currently consume relatively little high protein, low fat animal source foods that meet modern nutrition standards, such as dairy products and aquatic products (1). In China’s rural areas, agricultural production remains the primary source of income for rural households (32). Therefore, the agricultural commercialization of rural households in exchange for income will affect the market food purchase and nutritional status of residents.

With the commercialization of agriculture, a strong link has formed between smallholder production operations and agricultural markets in China. Currently, smallholder production and operations present an open situation. In addition to meeting their families’ food consumption needs, farming families increase their agricultural income by selling the agricultural products they produce. This increase in agricultural income represents an increase in disposable income, enabling farming families to purchase a wider variety of foods or increase the quantity of food bought in the market, thereby improving the dietary structure in terms of both quality and quantity. The increasing intake of food nutrients follows.

With the continuous improvement of the agricultural market system, farmers’ markets and other markets in rural areas are expanding, offering a greater variety of food types. This allows farmer households to purchase a diverse range of foods, including non-local and non-seasonal items (33). The study found that the interviewed farmer households generally visit the market every week, indicating that rural food purchases are very common in Nanjing and that the market is an essential means for residents to acquire diverse foods. The income derived from the commercialization of agricultural products is used to purchase other types of food in the market, thus indirectly impacting dietary diversity.

## 3 Data and methodology

### 3.1 Empirical research method

#### 3.1.1 Relevant concepts

Agriculture. As an agricultural powerhouse, agriculture plays an important role in the development of China’s national economy. However, there is no consensus among scholars on the definition of agriculture. The current understanding of agriculture is divided into two categories: narrow and broad agriculture. Narrow agriculture is limited to plantation, while broad agriculture encompasses four sectors: plantation, animal husbandry, forestry, and fishery (34). This study adopts the concept of broad agriculture.

Agricultural production diversity. Agricultural production diversity refers to the variety of food types produced by farmers within a specific time period (33). To assess agricultural production diversity, this paper quantifies it using simple crop counts and the Simpson Index (*SID*).

Simple Crop Count. In this study, using 1 year as the assessment criterion, simple crop counts (*N*) were obtained by summing the number of various crops grown, the number of aquatic species farmed, the number of types of livestock kept, and the number of types of trees planted by a farm household in the past year. The Simpson’s Index (*SID*) is often used in the field of ecology to assess the diversity of species or the diversity of organisms, and in agricultural production it is often used to measure the abundance (number of crops) and evenness (distribution of acreage) of agricultural products. Equation (1) is calculated as follows:


(1) $$SID=1-\overset{h}{\underset{h=1}{\sum }}p_{h}^{2}$$

*P_h_* is the proportion of the household’s cultivated area that is planted with each of the *h* crops. However, the interviews found that rural residents grow many kinds of vegetables each year, making it difficult to accurately determine the planting area of each vegetable. Therefore, all vegetables are considered as a whole and classified as a single crop.

Household dietary diversity score (*HDDS*). Few domestic surveys address the relationship between production diversity and dietary diversity in farmer households. Household level surveys are the more common choice for studies in this area (33). The *HDDS* is a widely used indicator to measure dietary diversity (13). It captures information on crop production diversity and household food consumption (7) and serves as an indicator of dietary nutrient adequacy (33). The *HDDS* is also an indicator of household food security and is usually measured as the number of food groups consumed over a 24-h or 7-day period (19). According to FAO, food is classified into 12 categories: cereals, potatoes, vegetables, fruits, meat, eggs, fish, pulses, dairy products, fats and oils, sugar or honey, and mixtures. *HDDS* is calculated as shown in Equation 2:


(2) $$\mathrm{HDDS}=\overset{12}{\underset{1}{\sum }}n$$

According to the farmers’ 24-h dietary review, if a food group was consumed during the 24-h period, then the n for that food group is 1, otherwise it will be 0. In addition, the same type of food will not be scored twice. The scores are then added up to calculate the household dietary diversity.

#### 3.1.2 Feasible generalized least squares (FGLS)

FGLS can correct the problems of heteroscedasticity, simultaneous correlation and sequence correlation caused by cross section data, and improve the consistency and effectiveness of parameter estimation. To analyze the impact of production diversity on dietary diversity, the econometric model of Equation (3) was constructed:


(3) $$\begin{array}{c} HDDS_{i}=\alpha_{0}+\alpha_{1}N_{i}+\alpha_{2}dfm_{i}+\alpha_{3}sc_{i}+\alpha_{4}tofp_{i}\\ +\alpha_{5}npfc_{i}+\alpha_{6}apc_{i}+\alpha_{7}\mathrm{ag}e_{i}+\alpha_{8}sf_{i}+\alpha_{9}\mathrm{lnap}i_{i}+\mu_{i} \end{array}$$

$HDDS_{i}$ is the dietary diversity of the i household $,N_{i}$ is the production diversity of the i household $,dfm_{i}$ is the distance from the nearest market of the i household $,sc_{i}$ is the sales channel of agricultural products of the i household $,tofp_{i}$ is the food purchased by the i household in the market $,npfc_{i}$ is whether there are new professional farmers in the i household $,apc_{i}$ is the agricultural product certification of the i household $,\mathrm{ag}e_{i}$ is the age of the respondents in the i household $,sf_{i}$ is the whether the i household is a small-scale farmers, $\mathrm{lnap}i_{i}$ is the agricultural production input of the i household, $\alpha_{0}\sim \alpha_{9}$ are the parameters to be estimated, and $\mu_{i}$ is the random error term of the model.

#### 3.1.3 Simultaneous equation model

In conjunction with the conceptual definitions in the previous section, this paper identifies the subject of the study as farm households engaged in broad agricultural production and management. This paper mainly discusses the interaction between agricultural production diversity and dietary diversity. On the one hand, the diversity of farmers’ agricultural production will promote the diversity of farmers’ diets. On the other hand, farmers’ demand for dietary diversity will also encourage farmers to plant more varieties of crops, thus promoting the diversity of farmers’ agricultural production. Considering the mutual causality between dietary diversity and production diversity, using a single equation model to test the effect of farmers’ production diversity on dietary diversity will bring about problems such as model error or endogeneity. The simultaneous equation model shows obvious superiority in dealing with the endogeneity problems which may be caused by the single equation model. Therefore, in this paper, we construct a set of equations composed of farmers’ dietary diversity equation and agricultural production diversity equation, and use simultaneous equations to explain the endogenous biochemical process and interaction between them. The formation of the complete simultaneous equation is shown in Equation (4):


(4) $$\left\{ \begin{array}{c} HDDS_{i}=\beta N_{i}+\gamma K_{i}+\varepsilon_{2}\\ N_{i}=\mathrm{\alpha HDD}S_{i}+\lambda J_{i}+\varepsilon_{1} \end{array}\right.$$

In the formula, *N_i_* represents the agricultural production diversity of sample *i*, *HDDS_i_* represents the dietary diversity of sample *i*, which are the core variables affecting the dietary diversity equation and the production diversity equation respectively; *β* is the coefficient of the variable to be estimated *N_i_*; *K_i_* is the control variable except *N* (representing agricultural production diversity) in *HDDS_i_*; *γ* is the coefficient of the variable to be estimated *K_i_*. *α* is the coefficient of the *HDDS_i_* variable to be estimated; *J_i_* is the control variable in *N_i_* other than *HDDS* (representing dietary diversity), and *λ* is the coefficient of the *J_i_* variable to be estimated; the error terms are denoted by ε *_1_* and ε *_2_*.

### 3.2 Dependent and independent variables

#### 3.2.1 Dependent and independent variables for dietary diversity equation

For the dietary diversity equation, the variable *HDDS* was used as the dependent variable and the following variables were used as independent variables.

##### 3.2.1.1 Explanatory variables

There is a positive correlation between farmers’ agricultural production diversity and dietary diversity (35). The more variety of food a farmer produces, the more he or she can increase the availability of agricultural resources within a certain range, whether the agricultural products are used for food consumption in the household or for commercialization, which is more likely to lead to increased dietary diversity in the farming household (33). Based on the research data in this paper, *N* and *SID* were selected to measure the diversity of farmers’ agricultural production.

##### 3.2.1.2 Control variables

Farmer characteristics are important factors that could influence household dietary diversity. Differences in age can lead to differences in dietary diversity (36, 37). Dietary diversity will be lower in people of middle and old age compared to young people. Referring to the WHO classification criteria, this paper classifies age into five age groups. Compared with traditional farmers, new professional farmers have higher cultural cultivation, know agricultural science and technology, and are good at management and administration (38), so they pay more attention to their dietary diversity and health. Compared with small-scale farmers, new agricultural management bodies have become the core force of rural agricultural laborers, have higher cultural literacy, and master cutting-edge agricultural science and technology knowledge and techniques (39), which helps to improve their dietary diversity. In this paper, whether to obtain a new type of professional farmers certificate as a new type of professional farmers as a measure.

Agricultural characteristics also influence household dietary diversity. Brand certification of agricultural products signifies the transformation of traditional agriculture into modern agriculture and helps to increase the premium price of agricultural products (40), which contributes to dietary diversity. In addition, agricultural production inputs contribute to increased production of agricultural products, which is important for food security. Studies have shown that an increase in the proportion of crop sales contributes to an increase in the dietary diversity of farmers (41). The certification standards of agricultural products taken into consideration in this paper include: pollution-free agricultural products certification, green food certification, organic agricultural products certification, and geographical indication agricultural products certification. Based on the data from the research of this paper, the sales channel of agricultural products was selected as an indicator to measure the commercialization of crops.

Market characteristics are also an important factor affecting household dietary diversity. In the absence of diversified subsistence production, the market plays a role in promoting dietary diversity, and distance to the nearest market is a key factor in the market factors affecting dietary diversity (42, 43). Whereas market food consumption is an important way to increase dietary diversity (44), the type of food purchased at the market was selected as a measure of market consumption based on the research data in this paper.

#### 3.2.2 Dependent and independent variables for production diversity equation

For the production diversity equation, the variable *N* and *SID* were used as dependent variables and the following variables were used as independent variables.

##### 3.2.2.1 Explanatory variables

Dietary diversity has the potential to influence farmers’ decision-making in crop production inversely by affecting their health status and human capital formation (45). Securing adequate dietary diversity can promote farmers’ health and human capital formation, which provides farmers with increased production diversity and further facilitates their production decision-making process.

##### 3.2.2.2 Control variables

Farm household characteristics are important factors that could influence production diversity. Relevant studies have found that differences in age and gender of farmers can lead to differences in cropping diversity, with females likely to be more motivated to diversify, while age may have both a facilitating and inhibiting effect on cropping diversity (46–49). Large-scale professional farmers are engaged in the primary production of a particular type of agricultural product and produce a single agricultural product (50).

Agricultural characteristics are also one of the important factors affecting household production diversity. Relevant studies have found that livestock operation has a driving effect on food production (51), which leads to the development of plantation (67) and thus promotes the production diversity of agriculture. Based on the actual situation of the questionnaire data in this study, the livestock income share (*poah*) was chosen to characterize the livestock operation. The degree of mechanization of arable land can affect the diversity of agricultural production by acting on the arable land replanting index and increasing the level of mechanization of arable land has a particularly significant impact on the improvement of the arable land replanting index of cash crops (52). Related studies have shown that areas with a higher degree of land fragmentation may be more suitable for diversified planting activities (48). Based on the questionnaire data of this study, we chose the degree of plot fragmentation (*pd*) as an indicator of the degree of land fine fragmentation.

Market characteristics affect household production diversity. The commercialization of agricultural products not only stimulates farmers’ enthusiasm for production, but also guides them to shift from the traditional mono-farming business model to a diversified business model (53, 54). Based on the reality of the questionnaire data in this study, the selection of agricultural product use (*ufa*) and market participation (*mp*) characterizes the commercialization of agricultural products. Food markets enable farmers to purchase the various types of food needed by farm families to compensate for the lack of self-sufficiency in agricultural production, and many agricultural products in food markets are cheaper to purchase than to produce, leading to some extent to a reduction in the diversity of agricultural production among farmers (44, 53, 55).

Policy characteristics are also important factors influencing household production diversity. The government’s agricultural production subsidy policy plays a positive role in increasing agricultural production motivation and promoting agricultural production and farmers’ incomes (56). Descriptive statistics of the variables are shown in Table 1.

**Table 1:** Definition of variables and descriptive statistics.

Variable	Variable symbol	Variable definition	Mean value	Standard deviation	Min	Max	Dietary equation	Production equation
Dependent variable								
Dietary diversity	HDDS	The type of food consumed by the household in the last 24 h household dietary diversity score	5.568	2.152	1	12	✓	
Production diversity 1: Number of crop types	N	The number of crops grown in the season + the number of aquatic products raised + the number of livestock raised + the number of tree species grown	2.267	1.507	0	8		✓
Production diversity 2: Simpson index	SID	Simpson index	0.228	0.285	0	1.189		✓
Independent variable								
Peasant household characteristics								
age	age	Age; 0 = 18–44 years old; 1 = 45–59 years old; 2 = 60–74 years old; 3 = 75–89 years old; 4 = 90 and above	1.578	0.814	0	4	✓	✓
sex	gender	Gender; 0 = female; 1 = male	0.618	0.487	0	1		✓
New type of professional farmer	npfc	Whether to obtain a new type of professional farmer certificate; 0 = no; 1 = yes	0.075	0.263	0	1	✓	
Small-scale farmer	sf	Whether they are small-scale farmers; 0 = no; 1 = yes	0 0.214	0.411	0	1	✓	
Large professional farmers	lpf	Whether it is a major professional breeding household, 0 = no; 1 = yes	0 0.071	0.258	0	1		✓
Agricultural characteristics								
Agricultural product certification	apc	The number of agricultural product certification types obtained by agricultural products	0.193	0.702	0	4	✓	
Sales channel of agricultural products	sc	Sales channels of agricultural products (number of types)	1.028	0.891	0	6	✓	
Agricultural production input	lnapi	Agricultural, forestry, animal husbandry and fishery production inputs of various agricultural products (such as the purchase of seeds, fertilizers, pesticides, aquatic fry, livestock calves, equipment, etc.) (yuan/year), logarithm	8.100	2.317	2.708	14.845	✓	
Share of income from animal husbandry	poah	Share of animal husbandry income (%)	2.336	11.820	0	100		✓
Mechanization degree of cultivated land	alm	Suitable degree of mechanization of cultivated land; 0 = most do not fit; 1 = about half fit; 2 = most fit	0 0.702	0.906	0	2		✓
Plot dispersion	pd	The dispersion degree of cultivated land; 0 = dispersed or more dispersed; 1 = general; 2 = more concentrated or concentrated	1.211	0.943	0	2		✓
Market characteristics								
Food purchased at the market	tofp	Number of types of food purchased at the market in the past week	5.506	2.723	1	12	✓	
Distance from the nearest market	dfm	Distance from home to nearest market (km)	2.404	2.299	0	7	✓	
Use of agricultural products	ufa	The use of agricultural products produced; 0 = eat half and sell half; 1 = eat more than half by yourself; 2 = more than half of sales	1.348	0.649	0	2		✓
Market participation	mp	Whether to sell agricultural products, 0 = no; 1 = yes	0 0.736	0.441	0	1		✓
Policy characteristics								
Government subsidies for agricultural production	gs	Number of types of government agricultural production subsidies enjoyed	0 0.736	0.928	0	5		✓

“✓” means selected as variable for the equation.

### 3.3 Study area

Nanjing is situated in a core area of urban agglomerations in the middle and lower reaches of the Yangtze River in China. It boasts abundant land and water resources, diverse landforms, suitable climate conditions, significant geographical advantages, and a rich cultural history with a strong agricultural production tradition. There are three main reasons for selecting Nanjing as the research site. Firstly, Nanjing has various forms of agricultural production. The city benefits from a robust supply of agricultural products and a favorable production environment, encompassing various sectors such as crop cultivation, animal husbandry, aquaculture, and forestry. The diversity reflects different agricultural development models and positions Nanjing as a stronghold of modern urban agriculture. Secondly, the rural population in Nanjing remains significant. By the end of 2022, Nanjing’s administrative divisions were divided into 11 districts, including 322 villagers’ committees. The number of permanent residents in the city is 9,491,100, while the rural population is 1,232,289. The urbanization rate is 87.01%. The city possesses a solid economic foundation and a relatively diverse dietary structure among farmers, making it representative of modern agricultural populations in China. Finally, Nanjing can represent the agricultural and dietary characteristics of China’s new first-tier cities. The city has not only developed its economic and agricultural infrastructure but has also implemented demonstration projects for agricultural modernization. It has successfully established modern agricultural demonstration zones and belts, becoming an important showcase for modern agriculture in China. This context provides a typical case for examining the influence of agricultural production diversity and market characteristics on dietary diversity, highlighting the relationship between agriculture and diet in contemporary urban settings.

### 3.4 Data sources

The data for this paper were obtained through a questionnaire survey of farm households. According to the relevant data of 2022 provided by Nanjing Bureau of Statistics, the neighborhood committees with cultivated land are screened out (as the basis for judging whether the neighborhood committees have farmers). According to the stratified random sampling strategy, the number of neighborhood committees with cultivated land is determined by the total number of neighborhood committees, which ensures the reliability and integrity of the samples. The research team conducted a survey on the production diversity and dietary diversity of farm households at the household level in Nanjing in September 2023, resulting in 318 valid questionnaires, achieving a 100 percent validity rate. The questionnaire collected information on farmers’ characteristics, household characteristics, production and operation characteristics, and market participation. The data such as the number of types of food consumed by farmers’ families were obtained by the method of 24-h dietary review. The set of crop types included four major categories: planted crops, farmed aquatic products, raised livestock, and planted trees, each subdivided by type. The survey methodology involved several steps.

Firstly, based on data provided by the Nanjing Municipal Bureau of Statistics in 2022, we screened neighborhood committees that retained arable land (used as a basis for determining whether there are farmers in the neighborhood committee). A total of 522 neighborhood committees were considered, excluding those in Qinhuai, Xuanwu, Gulou, Jianye, and Yuhuatai districts due to the lack of arable land, resulting in coverage of the remaining six districts.

Ensuring that all 6 district neighborhood committees were selected, we determined the number of neighborhood committees selected based on the total number of neighborhood committees with cultivated land according to the stratified random sampling strategy. When the total number of neighborhood committees was less than or equal to 10, 1 neighborhood committee was selected randomly. When the total number of neighborhood committees was greater than 10 and less than or equal to 30, 2 neighborhood committees were randomly selected. When the total number of neighborhood committees was greater than 30 and less than or equal to 50, 3 neighborhood committees were randomly selected. When the total number of neighborhood committees was greater than 50 and less than or equal to 80, 4 neighborhood committees were randomly selected. When the total number of neighborhood committees was greater than 80, 5 neighborhood committees were randomly selected. A total of 26 neighborhood committees were selected according to the total number of neighborhood committees in each district, and 320 samples were allocated to the selected neighborhood committees according to the proportion of households in each neighborhood committee. After rounding, 318 of the 320 questionnaires were distributed to villages (communities). Table 2 shows the sample distribution of the search area.

**Table 2:** Sample allocation.

Serial No.	District	Town/Street	Village/Community	Sample count
1	Qixia District	Longtan Street	Madou Village Committee	5
2	Qixia District	Bagua Island Street	Xiaba Village Committee	15
3	Jiangning District	Chunhua Street	Baishu Village Committee	10
4	Jiangning District	Hushu Street	Henan Community Committee	23
5	Jiangning District	Hengxi Street	Danyang Community Committee	19
6	Jiangning District	Tangshan Street	Luxi Village Committee	8
7	Jiangning District	Hengxi Street	Xinyang Community Committee	13
8	Pukou District	Tangquan Street	Quanxi Community Committee	11
9	Pukou District	Yongning Street	Youlian Village Committee	4
10	Pukou District	Yongning Street	Gaoli Community Committee	19
11	Pukou District	Pancheng Street	Duqiao Community Committee	7
12	Liuhe District	Jinniuhu Street	Heren Village Committee	15
13	Liuhe District	Ma’an Street	Huanggang Village Committee	16
14	Liuhe District	Chengqiao Street	Tanglou Community Committee	17
15	Liuhe District	Hengliang Street	Yuhuashi Village Committee	11
16	Liuhe District	Xiongzhou Street	Longhuying Village Committee	16
17	Lishui District	Zhetang Street	Qunli Community Committee	21
18	Lishui District	Jingqiao Town	Xiantan Village Committee	6
19	Lishui District	Honglan Street	Tangxi Village Committee	10
20	Lishui District	Shiqiao Street	Hengshan Village Committee	6
21	Lishui District	Jingqiao Town	Fengxiangling Community Committee	13
22	Gaochun District	Gucheng Street	Xingang Village Committee	13
23	Gaochun District	Qiqiao Street	Qiqiao Village Committee	13
24	Gaochun District	Yangjiang Town	Pingganwei Village Committee	14
25	Gaochun District	Yangjiang Town	Yongfeng Village Committee	5
26	Gaochun District	Gucheng Street	Huamiao Village Committee	8

## 4 Empirical results and analyses

### 4.1 Baseline regression results

The baseline model was estimated using Stata 8.0, and the results are presented in Table 3. The study’s findings indicate that the estimated coefficients of production diversity are significantly positive both before and after the inclusion of control variables. This suggests that production diversity has a significant positive effect on dietary diversity - the higher the level of production diversity, the greater the dietary diversity – which is consistent with previous research (33). According to the regression analysis using FGLS, a 1% increase in production diversity results in a 0.331 to 0.380% increase in dietary diversity.

**Table 3:** Baseline regression results of the impact of agricultural production diversity on the dietary diversity of farm households.

Variable	(1)	(2)	Multicollinearity
	FGLS	FGLS	
*N*	0.380*** (0.086)	0.331*** (0.070)	1.110 [0.905]
*dfm*		−0.134*** (0.037)	1.080 [0.929]
*sc*		0.208*(0.122)	1.280 [0.780]
*tofp*		0.335***(0.038)	1.040 [0.962]
*npfc*		0.793* (0.444)	1.090 [0.919]
*apc*		0.793*** (0.198)	1.170 [0.852]
*age*: *1*		−0.975** (0.422)	3.520 [0.284]
*age: 2*		−0.952** (0.417)	3.700 [0.270]
*age: 3*		−1.182** (0.489)	2.320 [0.432]
*age: 4*		−3.630*** (0.997)	1.060 [0.947]
*sf*		−0.476** (0.221)	1.040 [0.959]
*lnapi*		−0.045 (0.047)	1.450 [0.690]
Constant term	4.708*** (0.208)	4.263*** (0.610)	
Prob >*F*	0.000	0.000	
*R* ^2^	0.058	0.369	

Standard errors are listed in parentheses. * Significant at 10%; ** significant at 5%; *** significant at 1%. Values below the multicollinearity test are variance inflation factors; values in square brackets are tolerances.

From the perspective of control variables, the FGLS mean regression shows that both the number of types of food purchased at the market in the past week and the number of types of produce certified for agricultural products have a statistically significant positive effect on the dietary diversity of farm households at the 1 percent level. This means that the more types of food purchased in the market in the past week, the higher the dietary diversity of the farmers, highlighting that market purchases are indeed an important way to increase dietary diversity. Additionally, the greater the number of certified types of agricultural products produced, the more importance farmers place on agricultural production knowledge, thereby increasing their consciousness of improving dietary diversity, especially among those with high levels of dietary diversity.

### 4.2 Robustness tests

Substitution of core variables and models was used to ensure the robustness of the previous results. Referring to existing studies, Simpson’s Diversity Index (SID) replaced simple crop counts (N) as a means of assessing the level of crop production diversity, the core explanatory variable. Based on the type of data for the explanatory variables, we chose an ordered Logit model to analyze the data. Table 4 shows the results of the tests for the replacement variables and the model.

**Table 4:** Robustness tests results.

Variable	(1)	(2)	(3)
	FGLS	Logit	Marginal utility
*SID*	1.290*** (0.356)	1.390*** (0.386)	4.016*** (1.552)
*dfm*	−0.138*** (0.038)	−0.114** (0.046)	0.892** (0.041)
*sc*	0.176 (0.124)	0.245* (0.127)	1.278* (0.162)
*tofp*	0.340*** (0.039)	0.410*** (0.046)	1.506*** (0.070)
*npfc*	0.999** (0.452)	1.140*** (0.441)	3.127*** (1.379)
*apc*	0.809*** (0.202)	1.108*** (0.186)	3.027*** (0.563)
*age: 1*	−0.888** (0.430)	−1.184*** (0.405)	0.306*** (0.124)
*age: 2*	−0.953** (0.426)	−1.228*** (0.405)	0.293*** (0.119)
*age: 3*	−1.235** (0.497)	−1.332** (0.521)	0.264** (0.138)
*age: 4*	−3.966*** (1.019)	−4.651*** (1.639)	0.010*** (0.016)
*sf*	−0.415* (0.226)	−0.475* (0.248)	0.622* (0.154)
*lnapi*	−0.034 (0.048)	−0.042 (0.057)	0.959 (0.055)
Constant term	4.599*** (0.610)		
Prob >F	0.000		
*R* ^2^	0.348		
Pseudo *R*^2^		0.121	0.121

Standard errors are listed in parentheses. * Significant at 10%; ** significant at 5%; *** significant at 1%.

Regression 1 in Table 4 presents the estimated results after replacing the core variable *N*. The coefficient of *SID* is the same in both sign and significance as in the original baseline regression, meaning that the positive impact of production diversity on dietary diversity is relatively reliable. Regression 2 shows the results after replacing N and the regression model, while regression 3 provides the estimation of its marginal effect. Even with the replacement of models and variables, production diversity continues to have a significant positive effect on dietary diversity.

### 4.3 Endogeneity treatment

Using the Hausman test, it was found that the *p*-value is less than 0.1, leading to the rejection of the null hypothesis. This result indicates the presence of an endogeneity problem. To further enhance the robustness of the study, Ordinary Least Squares (OLS), Two-Stage Least Squares (2SLS), Three-Stage Least Squares (3SLS), and iterative 3SLS were employed for analysis. The *p*-values of the F-tests are all 0.000, indicating that the model setting is appropriate and has joint significance. Notably, the 3SLS and OLS estimation coefficients differ, so the results of 3SLS are primarily analyzed.

As shown by the 3SLS estimation results in Table 5, in the influence of production diversity on dietary diversity, each increase of 1% in production diversity leads to an increase of 0.406% in dietary diversity. With each increase of 1% in the distance to the nearest market, dietary diversity decreases by 0.079%. Each increase of 1% in agricultural product sales channels leads to an increase of 0.256% in dietary diversity. Each increase of 1% in food purchased in the market leads to an increase of 0.334% in dietary diversity. From the results, it can be seen that the influence of agricultural product production input on dietary diversity has a negative effect. With each increase of 1% in agricultural product production input, dietary diversity decreases by 0.014%, but the result is not significant.

**Table 5:** Estimation results of simultaneous equation models OLS, 2SLS, 3SLS, and 3SLS ~ r.

Variable	(1)	(2)	(3)	(4)
	OLS	2SLS	3SLS	3SLS ~ r
*HDDS*				
*N*	0.320*** (0.071)	0.405*** (0.151)	0.406*** (0.147)	0.406*** (0.147)
*dfm*	−0.094** (0.045)	−0.095** (0.045)	−0.079* (0.043)	−0.076* (0.042)
*sc*	0.261** (0.131)	0.250* (0.132)	0.256** (0.124)	0.256** (0.123)
*tofp*	0.333*** (0.039)	0.332*** (0.039)	0.334*** (0.038)	0.334*** (0.038)
*npfc*	0.534 (0.405)	0.450 (0.427)	0.730* (0.401)	0.772* (0.397)
*apc*	0.985*** (0.146)	0.973*** (0.147)	0.925*** (0.142)	0.918*** (0.141)
*age:1*	−1.257*** (0.388)	−1.295*** (0.394)	−1.288*** (0.384)	−1.287*** (0.385)
*age:2*	−1.294*** (0.388)	−1.317*** (0.390)	−1.331*** (0.381)	−1.332*** (0.382)
*age:3*	−1.139** (0.493)	−1.085** (0.501)	−1.088** (0.487)	−1.087** (0.488)
*sf*	−0.421* (0.255)	−0.416 (0.256)	−0.483** (0.240)	−0.491** (0.237)
*lnapi*	−0.015 (0.052)	−0.004 (0.055)	−0.014 (0.052)	−0.015 (0.052)
_cons	4.076*** (0.663)	3.829*** (0.768)	3.853*** (0.737)	3.812*** (0.734)
*R*-squared	0.440	0.437	0.435	0.435
*N*				
*HDDS*	0.177*** (0.043)	0.175* (0.089)	0.191** (0.086)	0.193** (0.086)
*age:1*	0.594* (0.306)	0.592* (0.317)	0.590* (0.307)	0.589* (0.307)
*age:2*	0.633** (0.309)	0.630* (0.327)	0.603* (0.316)	0.599* (0.316)
*age:3*	0.081 (0.376)	0.079 (0.383)	0.059 (0.370)	0.056 (0.370)
*gender*	0.275 (0.175)	0.275 (0.176)	0.263 (0.162)	0.261 (0.160)
*ufa:1*	0.145 (0.303)	0.144 (0.304)	0.081 (0.280)	0.071 (0.277)
*ufa:2*	−0.690** (0.278)	−0.691** (0.284)	−0.823*** (0.265)	−0.843*** (0.262)
*tofp*	−0.070** (0.034)	−0.070* (0.042)	−0.078* (0.041)	−0.079* (0.041)
*poah*	0.017** (0.007)	0.017** (0.007)	0.018*** (0.006)	0.018*** (0.006)
*alm:1*	0.526* (0.317)	0.527* (0.318)	0.530* (0.294)	0.531* (0.291)
*alm:2*	0.711*** (0.197)	0.712*** (0.199)	0.658*** (0.186)	0.650*** (0.184)
*pd:1*	−0.110 (0.364)	−0.111 (0.367)	−0.198 (0.339)	−0.211 (0.335)
*pd:2*	−0.404** (0.183)	−0.404** (0.184)	−0.339** (0.170)	−0.399** (0.168)
*lpf*	−0.717* (0.372)	−0.716* (0.373)	−0.677* (0.346)	−0.674** (0.342)
*mp*	0.534** (0.255)	0.535** (0.264)	0.588** (0.246)	0.596** (0.243)
*gs*	0.231** (0.091)	0.231** (0.091)	0.199** (0.084)	0.194** (0.083)
cons	0.710 (0.583)	0.718 (0.687)	0.784 (0.654)	0.795 (0.652)
*R*-squared	0.311	0.311	0.309	0.308
observations	318	318	318	318

Standard errors are listed in parentheses. * Significant at 10%; ** significant at 5%; *** significant at 1%.

In the influence of dietary diversity on production diversity, each increase of 1% in dietary diversity leads to an increase of 0.191% in production diversity. Compared with half of the agricultural products being used for self-consumption and half for sales, when more than half are for sales, production diversity decreases by 0.823%, indicating that a high level of agricultural commercialization promotes agricultural specialization to a certain extent. Each increase of 1% in food purchased in the market leads to a decrease of 0.078% in production diversity, suggesting that market purchases have a substitution effect on farmers’ production. Compared with farmers with fragmented cultivated land, farmers with concentrated cultivated land have a 0.339% decrease in production diversity, indicating that overly concentrated cultivated land is not conducive to farmers’ development of high production diversity.

The 3SLS estimation results of the simultaneous equation model and FGLS estimates are consistent in terms of the estimation sign and significance of the explanatory variables, but the numerical values of the estimation coefficients have changed. For the core variable of agricultural production diversity, there is a certain gap between the core variable coefficient value (0.406) estimated by 3SLS and the core variable coefficient value (0.331) estimated by FGLS. It can be seen that due to the influence of endogeneity problems, the estimation result of the baseline regression FGLS underestimates the positive impact of agricultural production diversity on dietary diversity.

After addressing the endogeneity problem, it is evident that production diversity and dietary diversity have a bidirectional promoting effect. The simultaneous equation model demonstrates that the influence coefficients of production diversity on dietary diversity and vice versa are significantly positive, indicating a mutual enhancement between the two. There is a positive synergistic growth effect between agricultural production diversity and dietary diversity, where they reinforce each other, forming a virtuous cycle. The results are significant, highlighting the importance of developing agricultural production diversity while promoting dietary diversity. Increasing agricultural production diversity can improve the overall nutritional status of farming families by providing them with more dietary choices. Simultaneously, enhancing dietary diversity will encourage the diversification of agricultural production.

### 4.4 The mediation effect of agricultural production diversity on market participation and dietary diversity

Based on the benchmark regression results, robustness tests, and the outcomes from the simultaneous equation model discussed previously, it is evident that production diversity positively influences dietary diversity. This section further explores whether production diversity serves as a mediator in the relationship between market participation and the dietary diversity of rural residents. The analysis employs 500 replicate samplings with a 95% confidence interval, and the specific findings are detailed in Table 6.

**Table 6:** Results of Bootstrap mediation effect.

	Coef	Bias	Bootstrap Std err	95% conf. Interval	
Indirect effect	0.164	−0.005	0.091	0.010–0.343	(P) (BC)
				0.020–0.413	
Direct effect	0.432	−0.001	0.321	−0.240–1.053	(P) (BC)
				−0.265–1.048	
Total effect	0.596	−0.006	0.328	−0.085–1.239	(P) (BC)

The research findings indicate that the confidence interval of the mediating effect does not include zero, confirming its validity. This suggests that production diversity mediates the relationship between market participation and dietary diversity. Moreover, the confidence interval of the direct effect contains zero, indicating a complete mediation. Thus, the influence of market participation on dietary diversity is fully realized through production diversity.

Firstly, market participation acts as an external factor that encourages farmers to pay closer attention to market demands for various agricultural products. The level of market prices serves as a crucial indicator, with price fluctuations directly influencing farmers’ production decisions. When market prices for certain crops increase, farmers may be inclined to focus on high-yield monocultures, thereby reducing production diversity. Conversely, if the price mechanism encourages diversification (such as price support for some crops or local varieties), it promotes production diversity. Farmers involved in the market tend to cultivate a broader range of agricultural products to meet the evolving market demands, which promotes agricultural production diversity and enriches the types and scope of agricultural product supply. Secondly, agricultural production diversity has a direct influence on the dietary diversity of rural households. With the improvement of the agricultural market, more farmers commercialize their agricultural products. This commercialization allows farmers to access a greater variety of food types, providing them with more options and enabling them to consume a wider range of foods, thereby enhancing dietary diversity. Thirdly, production diversity has an indirect effect on dietary diversity. As a key determinant of food availability, market access can significantly influence dietary diversity. One common indicator of market access is the geographical distance between farmers and the nearest marketable food market. Studies have shown that households located in remote areas often exhibit lower dietary diversity (13). High levels of market access enable farmers to more easily sell their products, fostering a shift toward market-oriented farming. This market engagement allows farmers to earn income and purchase a wider variety of foods, enhancing their dietary diversity. In response to market demands, farmers may diversify their agricultural production, sell a broader range of products, and thereby increase their agricultural income, which strengthens their economic capacity. Therefore, by shortening distances for better access to markets, it may help to improve dietary diversity.

Market participation influences the dietary diversity of rural households by affecting agricultural production diversity. The existence of mediation effect demonstrates the significant role of market mechanisms in promoting dietary diversity among rural households and emphasizes the impact of agricultural production diversity on dietary diversity.

## 5 Discussion

The empirical research of this paper is carried out through four main parts: Firstly, the feasible generalized least square method is used to study how the diversity of agricultural production affects the dietary diversity of farmers; Secondly, the core variable, dependent variable and econometric model are replaced for robustness test. Thirdly, we use Hausman test to test the endogeneity problem of the model, and adopt the simultaneous equation model estimation method to solve the endogeneity problem of the model. Finally, we explore the mediating role of productive diversity in the influence of market participation on dietary diversity.

### 5.1 The influence of agricultural production diversity and market purchases on dietary diversity

The research findings indicate a significant positive correlation between agricultural production diversity and household-level dietary diversity. The dietary diversity of farmer households relies on their own agricultural production, highlighting the self-sufficiency characteristics of farmers in Nanjing in terms of food consumption, which aligns with the current production status of farmers in China (57). The survey reveals that the majority of farmer households cultivate several types of crops preferred by family members in their front and back yards, confirming the close relationship between agricultural production diversity and dietary diversity. Although a significant positive correlation exists between the two (15), the results indicate that the coefficient of agricultural production diversity is relatively small. The slight positive correlation between agricultural production diversity and household dietary diversity has also been corroborated by other studies (5, 58). Empirical results suggest that to increase household dietary diversity by one type, agricultural production diversity needs to increase by three types. Therefore, to improve household dietary diversity, it may be essential to substantially increase agricultural production diversity to effectuate an impact on dietary diversity.

Although market purchases are positively correlated with dietary diversity, the strength of this correlation is weaker than that of agricultural production diversity. With economic development, the continuous improvement of infrastructure (59), such as rural markets, facilitates farmers’ access to food markets. Consequently, smallholder farmers are no longer fully self-sufficient. Instead, they come to rely on agricultural markets for meat, seafood, oilseeds, and even vegetables (60). On the other hand, the agricultural products produced by farmer households are not exclusively used for personal consumption (61). A portion may be given to friends and relatives or sold in agricultural markets to generate income (62). This shift leads to a smaller coefficient of agricultural production diversity, reflecting changes in the farming household production model and an increase in the commercialization of agricultural products. Although both production diversity and market purchases are important ways for enhancing dietary diversity, the research findings indicate that the positive impact of production diversity on dietary diversity is greater than that of food purchased in the market.

In most rural areas of China, dietary diversity among farmer households primarily relies on agricultural production. Although rural infrastructure has improved and agricultural markets increasingly facilitate access to a variety of food items (62), in economically developed regions like Nanjing, agricultural production diversity still depends more on their own production rather than market purchases. This suggests that in many parts of China, particularly in regions with less economic development than Nanjing, dietary diversity in rural households continues to be primarily derived from their own agricultural production rather than market purchases (63). Enhancing agricultural production diversity to improve dietary diversity among farmer households is thus more nutritionally meaningful and provides better food security (64). Due to disparities in urban and rural development and economic constraints, the types and quantities of food that rural residents can purchase in the market may be limited (65), resulting in a greater impact of production diversity on dietary diversity.

### 5.2 The influence of other factors on dietary diversity

The results indicate that possessing a new professional farmer certificate significantly and positively impacts dietary diversity, while age and small-scale farmers’ status significantly and negatively impact dietary diversity. Agricultural characteristics such as sales channels and certifications of agricultural products also significantly affect dietary diversity. Market characteristics, including the distance to the nearest market and food purchased in the market, have significant impacts on dietary diversity. The farther a household is from the nearest market, the lower its dietary diversity, indicating that remoteness restricts access to fresh and diverse foods, thereby limiting food choices and reducing dietary diversity. Generally, dietary diversity of farmer households can be improved by shortening the distance to markets and enhancing market infrastructure (9). However, empirical analysis shows that a 1% increase in the distance to the nearest market results in a 0.079% decrease in dietary diversity, suggesting that the effect of reducing distance or expanding market infrastructure on improving dietary diversity is limited.

## 6 Conclusions and policy recommendations

This study examines farmer households by collecting 318 sets of data through field research in Nanjing. Methods such as feasible generalized least squares and simultaneous equation models were employed to address data heteroscedasticity and endogeneity, exploring the relationship between production diversity and dietary diversity among farmer households in Nanjing. The research yielded the following conclusions: Firstly, agricultural production has shifted from a “small and comprehensive” approach to specialization. This paper uses the additive crop count of agriculture, forestry, animal husbandry and fishery industries as the measurement index of agricultural production diversity, and carries out multiple evaluations on the production diversity of rural residents in Nanjing. The results show that the production diversity index of Nanjing City was concentrated in the range of 1 to 3, with an average value of 2.324, indicating that the agricultural production diversity of Nanjing City was low. The low agricultural production diversity in Nanjing reflects this trend in China, where agriculture is transitioning from “small and comprehensive” to specialized production. In Nanjing, the planting industry focuses on grains and vegetables, animal husbandry primarily involves poultry, aquatic farming centers on fish and crustaceans, and forestry mainly consists of economic forests. Secondly, household dietary diversity is low, and the food consumption structure is imbalanced. The dietary diversity of rural residents in Pukou, Lishui, and Gaochun Districts is higher than the average in Nanjing, whereas it is lower in Qixia, Jiangning, and Luhe Districts. In Nanjing, the consumption of high-nutritional value foods such as aquatic products and dairy products constitutes only 5.295 and 3.344%, respectively, highlighting the unbalanced food consumption structure of farmer households. Thirdly, agricultural production diversity significantly positively impacts dietary diversity. Higher levels of agricultural production diversity correspond to higher dietary diversity among farmer households. Fourthly, there is a differential impact of production diversity and market purchases on dietary diversity. The empirical analysis reveals that a greater variety of market-purchased food in the past week corresponds to higher dietary diversity levels among farmers. Market-purchased food is indeed crucial for enhancing dietary diversity. However, after addressing the endogeneity issue, it is evident that the correlation between market-purchased food and dietary diversity is weaker compared to production diversity. This suggests that while both production diversity and market-purchased food are vital for increasing dietary diversity, production diversity has a more substantial positive effect. Additionally, the simultaneous equation model analysis indicates that increased market purchases significantly negatively impact agricultural production diversity, implying a substitution effect of market purchases on farmer production.

Based on the above research conclusions, this article draws the following policy implications: Firstly, the study found that rural residents in Nanjing commonly use the land around their homes for self-production and self-consumption of agricultural activities. To safeguard dietary diversity and food security for Nanjing’s farmer households, the development and utilization of this land should be improved. Agricultural activities can be carried out in these spaces to enhance self-sufficiency in food consumption and dietary diversity. Additionally, inter-cropping and set-cropping methods should be employed to improve land resource utilization quality and efficiency. Secondly, improving the agricultural market and enriching the food supply are crucial. The food consumed by farmers’ families originates from agricultural production, market supply, and gifts from relatives and friends, with market supply playing a significant role in affecting farmers’ food consumption. Strengthening the construction of diversified and multi-functional agricultural markets is essential to improving rural living conditions and building beautiful villages. Agricultural markets should facilitate easy access to fresh food, provide a variety of food options, and meet the dietary needs of rural residents. The development of these markets should consider the traffic situation and terrain of rural areas, conduct in-depth analyses of the distribution of agricultural markets, and prioritize improvements in areas with more challenging conditions. Integrating local characteristics into market construction and supporting the establishment of milk source bases will ensure a wide variety of food for rural residents and increase the proportion of high-nutritional-value foods, thereby improving their nutritional structure and ensuring food security. Thirdly, strengthening farmer training and improving nutrition knowledge is essential. Many rural areas face issues such as unscientific food consumption patterns, low dietary diversity, and insufficient nutrition. To achieve sustainable food consumption and support the implementation of the Healthy China strategy, it is necessary to build a strong conceptual defense for nutritional and food security. Educating rural residents on the importance of developing reasonable dietary habits is vital. The empirical results indicate that new professional farmers exhibit higher dietary diversity, highlighting the significance of farmers with modern concepts, qualities, and abilities. Therefore, it is important to enhance nutrition knowledge training in rural areas, cultivate new professional farmers, and conduct educational activities to raise awareness of dietary health. Actively responding to <The notice of the General Office of the Ministry of Agriculture and Rural Affairs on Improving the Cultivation of High-quality Farmers in 2024> issued by China, comprehensive literacy courses are offered. Popularize knowledge, concepts and advocacy requirements in the fields of policies, laws and regulations related to agriculture, rural areas and agriculture, green agricultural development, agricultural standardization, quality and safety of agricultural products, and protection and construction of cultivated land. Publicizing dietary nutrition knowledge will also improve the food consumption structure of rural residents, cultivate high-quality farmers, and help them develop correct scientific dietary concepts and healthy eating habits. This will promote sustainable food consumption and the implementation of the Healthy China strategy.

This research has some limitations. Firstly, some social factors that were not taken into account in the study could have influenced the results, such as education level, cultural practices and the absence of information on gender dynamics within households. Secondly, the time span is insufficient, and the cross-section data lacks panel data, which shows the characteristics of individual changes over time, so the long-term dynamic evolution of crop production diversity and farmers’ dietary diversity cannot be observed. Thirdly, seasonal effects need further consideration. In most regions of China, summer is the peak season for agricultural production, with production diversity reaching its highest levels. The research and corresponding data in this study are primarily concentrated in the summer period, which provides a basis for horizontal comparisons. Additionally, the survey revealed that many areas utilize plastic sheds for greenhouse cultivation, which enhances agricultural diversity across different seasons. Greenhouse planting systems can, to some extent, mitigate the seasonal constraints on the diversity of agricultural products, such as vegetables, thereby reducing the overall impact of seasonality on production diversity. However, due to limitations in data collection, the impact of seasonal variations has not been thoroughly considered, which will be a key focus for future research. In the future research process, farmers’ production mode and distance from the city can be taken into account in sample selection to ensure that the selected samples can represent the average situation of the region, further explore the reasons for the differences between regions, expand the time span of data, consider seasonal effects, and obtain panel data through investigation to improve the research quality.

## Data Availability

The raw data supporting the conclusions of this article will be made available by the authors, without undue reservation.

## References

[1] Shen Q Zhong T. Did household income loss have an immediate impact on animal-source foods consumption during the early Stage of the COVID-19 pandemic? Food Secur. (2023) 12:1424. doi: 10.3390/foods12071424, PMID: 37048245 PMC10093368

[2] Liu Y.. Report on nutrition and chronic disease status of chinese residents. Chin. J. Clin. Nutr. (2020) 42:521.

[3] Maxwell D Vaitla B Coates J. How do indicators of household food insecurity measure up? An empirical comparison from Ethiopia. Food Policy. (2014) 47:107–16. doi: 10.1016/j.foodpol.2014.04.003

[4] FAO. Rome declaration on world food security and world food summit plan of action. Injury Prevention. (1996) 12:163–81.

[5] Jones AD Shrinivas A Bezner-Kerr R. Farm production diversity is associated with greater household dietary diversity in Malawi: findings from nationally representative data. Food Policy. (2014) 46:1–12. doi: 10.1016/j.foodpol.2014.02.001

[6] Headey D Hirvonen K Hoddinott J Stifel D. Rural food markets and CHILD nutrition. Am J Agric Econ. (2019) 101:1311–27. doi: 10.1093/ajae/aaz032, PMID: 33303995 PMC7722321

[7] Carletto C Corral P Guelfi A. Agricultural commercialization and nutrition revisited: empirical evidence from three African countries. Food Policy. (2017) 67:106–18. doi: 10.1016/j.foodpol.2016.09.020, PMID: 28413250 PMC5384450

[8] Torheim LE Ouattara F Diarra MM Thiam FD Barikmo I Hatloy A. Nutrient adequacy and dietary diversity in rural Mali: association and determinants. Eur J Clin Nutr. (2004) 58:594–604. doi: 10.1038/sj.ejcn.1601853, PMID: 15042127

[9] Islam AHMS von Braun J Thorne-Lyman AL Ahmed AU. Farm diversification and food and nutrition security in Bangladesh: empirical evidence from nationally representative household panel data. Food Secur. (2018) 10:701–20. doi: 10.1007/s12571-018-0806-3

[10] Sariyev O Loos TK Khor LY. Intra-household decision-making, production diversity, and dietary quality: a panel data analysis of Ethiopian rural households. Food Secur. (2021) 13:181–97. doi: 10.1007/s12571-020-01098-9

[11] Kadiyala S Harris J Headey D Yosef S Gillespie S. Agriculture and nutrition in India: mapping evidence to pathways In: Dube L Webb P Arora NK Pingalid P, editors. Paths of convergence for agriculture, health, and wealth, vol. 1331 (2014). 43–56. 10.1111/nyas.12477 25098622

[12] Ecker O. Agricultural transformation and food and nutrition security in Ghana: does farm production diversity (still) matter for household dietary diversity? Food Policy. (2018) 79:271–82. doi: 10.1016/j.foodpol.2018.08.002

[13] Sibhatu KT Krishna VV Qaim M. Production diversity and dietary diversity in smallholder farm households. Proc Natl Acad Sci. (2015) 112:10657–62. doi: 10.1073/pnas.1510982112, PMID: 26261342 PMC4553771

[14] Sibhatu KT Qaim M. Farm production diversity and dietary quality: linkages and measurement issues. Food Secur. (2018) 10:47–59. doi: 10.1007/s12571-017-0762-3

[15] Yan Z Xiao XY Jiao JJ Lin W. How does agricultural production diversity nourish household dietary diversity? Evidence from China. Glob Food Secur Agric Policy Econ Environ. (2024) 40:100749. doi: 10.1016/j.gfs.2024.100749, PMID: 40226602

[16] Guo X Zeng X Wang Q Luo X. The structural division of smallholder farmers in China: an analytical framework based on smallholder farmer survey data in Sichuan province. Chin Rural Econ. (2018) 40:76–88106.

[17] Ruel MT Quisumbing AR Balagamwala M. Nutrition-sensitive agriculture: what have we learned so far? Glob Food Secur Agric Policy Econ Environ. (2018) 17:128–53. doi: 10.1016/j.gfs.2018.01.002

[18] Koppmair S Kassie M Qaim M. Farm production, market access and dietary diversity in Malawi. Public Health Nutr. (2017) 20:325–35. doi: 10.1017/s1368980016002135, PMID: 27609557 PMC5244442

[19] Romeo A Meerman J Demeke M Scognamillo A Asfaw S. Linking farm diversification to household diet diversification: evidence from a sample of Kenyan ultra-poor farmers. Food Secur. (2016) 8:1069–85. doi: 10.1007/s12571-016-0617-3

[20] Jones AD. Critical review of the emerging research evidence on agricultural biodiversity, diet diversity, and nutritional status in low-and middle-income countries. Nutr Rev. (2017) 75:769–82. doi: 10.1093/nutrit/nux040, PMID: 29028270 PMC5914317

[21] Gibson J Rozelle S. How elastic is calorie demand? Parametric, nonparametric, and semiparametric results for urban Papua New Guinea. J Dev Stud. (2002) 38:23–46. doi: 10.1080/00220380412331322571

[22] Huang KS Gale F. Food demand in China: income, quality, and nutrient effects. China Agric Econ Rev. (2009) 1:395–409. doi: 10.1108/17561370910992307

[23] Tian X Yu X. The demand for nutrients in China. Front Econ China. (2013) 8:186–206.

[24] Cockx L Colen L De Weerdt J. From corn to popcorn? Urbanization and dietary change: evidence from rural-urban migrants in Tanzania. World Dev. (2018) 110:140–59. doi: 10.1016/j.worlddev.2018.04.018

[25] Sinyolo S Murendo C Nyamwanza AM Sinyolo SA Ndinda C Nwosu CO. Farm production diversification and dietary diversity among subsistence farming households: panel data evidence from South Africa. Sustain For. (2021) 13:10325. doi: 10.3390/su131810325, PMID: 40225413

[26] Pall Z Perekhozhuk O Glauben T Prehn S Teuber R. Residual demand measures of market power of Russian wheat exporters. Agric Econ. (2014) 45:381–91. doi: 10.1111/agec.12072

[27] Mulenga BP Ngoma H Nkonde C. Produce to eat or sell: panel data structural equation modeling of market participation and food dietary diversity in Zambia. Food Policy. (2021) 102:102035. doi: 10.1016/j.foodpol.2021.102035

[28] Zhang L Luo B. How can small farmers be incorporated into modern agricultural development? Evidence from wheat-producing areas of China. Econ Res J. (2018) 53:144–60.

[29] Kiptoo E Waswa LM Ayuya OI. Linking farm production to household diets: evidence from two low potential areas in Kenya. Cogent Food Agric. (2021) 7:1913842. doi: 10.1080/23311932.2021.1913842

[30] Zhu S Yang R Lu W Wu B. Rural land transfer and the change of agricultural production mode in China. J Manag World. (2024) 40:7–21. doi: 10.19744/j.cnki.11-1235/f.2024.0011

[31] Chegere MJ Stage J. Agricultural production diversity, dietary diversity and nutritional status: panel data evidence from Tanzania. World Dev. (2020) 129:104856. doi: 10.1016/j.worlddev.2019.104856, PMID: 40226602

[32] Kabir MR Halima O Rahman N Ghosh S Islam MS Rahman H. Linking farm production diversity to household dietary diversity controlling market access and agricultural technology usage: evidence from Noakhali district, Bangladesh. Heliyon. (2022) 8:e08755. doi: 10.1016/j.heliyon.2022.e08755, PMID: 35071816 PMC8762389

[33] Huang Z.-Y. Sun J.-M. Guo Y.-Z. Wang X.-L. Ma Y.-Q. The influence of agricultural production diversity of farmers on their Dietary diversity and nutritional health. Scientia Agricultura Sinica. (2019) 52:3108–3121.

[34] Luo H. Research on domestic agricultural carbon source/sink effect: perspectives, advances and improvements. Acta Ecol Sin. (2022) 42:3832–41.

[35] Oyarzun PJ Mary Borja R Sherwood S Parra V. Making sense of agrobiodiversity, diet, and intensification of smallholder family farming in the Highland Andes of Ecuador. Ecol Food Nutr. (2013) 52:515–41. doi: 10.1080/03670244.2013.769099, PMID: 24083517

[36] Bandyopadhyay A Haile B Azzarri C Some J. Analyzing the drivers of household dietary diversity: evidence from Burkina Faso. Food Nutr Bull. (2021) 42:530–50. doi: 10.1177/03795721211029092, PMID: 34467801 PMC8637355

[37] Rupa JA Umberger WJ Zeng D. Does food market modernisation lead to improved dietary diversity and diet quality for urban Vietnamese households? Aust J Agric Resour Econ. (2019) 63:499–520. doi: 10.1111/1467-8489.12308

[38] Yang X Qi Z Yang C Liu Z. Can the new type of agricultural management promote the promotion of ecological agricultural technology: take Rice and shrimp co-cultivation technology as an example. Resour Environ Yangtze Basin. (2021) 30:2545–56.

[39] Zhang B Zhang L Fu Z Wang J. The adoption behaviors and the influencing factors of water saving irrigation technology by new agricultural management entities: a case study of Beijing. Res Agric Modern. (2017) 38:987–94.

[40] Fan L. Wang P. Zhang J. (2020). Temporal and spatial distribution characteristics of geographical indication agricultural products and their influencing factors. World Agriculture. (2020). 120–7. doi: 10.13856/j.cn11-1097/s.2020.02.014

[41] Otekunrin OA Otekunrin OA. Exploring dietary diversity, nutritional status of adolescents among farm households in Nigeria: do higher commercialization levels translate to better nutrition? Nutr Food Sci. (2023) 53:500–20. doi: 10.1108/nfs-03-2022-0104

[42] Chegere MJ Kauky MS. Agriculture commercialisation, household dietary diversity and nutrition in Tanzania. Food Policy. (2022) 113:102341. doi: 10.1016/j.foodpol.2022.102341, PMID: 40226602

[43] Nyantakyi-Frimpong H. Agricultural diversification and dietary diversity: a feminist political ecology of the everyday experiences of landless and smallholder households in northern Ghana. Geoforum. (2017) 86:63–75. doi: 10.1016/j.geoforum.2017.09.003

[44] Bernzen A Mangnus E Sohns F. Diversify, produce or buy? An analysis of factors contributing to household dietary diversity among shrimp and non-shrimp farmers in coastal Bangladesh. Food Secur. (2022) 14:741–61. doi: 10.1007/s12571-021-01245-w, PMID: 35106101 PMC8795731

[45] Alderman H. The response of Child nutrition to changes in income: linking biology with economics. CESifo Econ Stud. (2012) 58:256–73. doi: 10.1093/cesifo/ifs012

[46] Benin S Smale M Pender J Gebremedhin B Ehui S. The economic determinants of cereal crop diversity on farms in the Ethiopian highlands. Agric Econ. (2004) 31:197–208. doi: 10.1016/j.agecon.2004.09.007

[47] Drescher L. S. Goddard E. W. (2008). Observing changes in Canadian demand for food diversity over time. Access & Download Stats. (2008). doi: 10.22004/AG.ECON.6357

[48] Yan M Zhong T. The difference of crop diversity between migrant professional farmers and local peasant households and its influencing factors. Resour Sci. (2018) 40:1752–61.

[49] Zawedde BM Harris C Alajo A Hancock J Grumet R. Factors influencing diversity of Farmers' varieties of sweet potato in Uganda: implications for conservation. Econ Bot. (2014) 68:337–49. doi: 10.1007/s12231-014-9278-3

[50] Jiang J Zhang S Li B Ding Z. Does blindness exist in the farmers' intention to expand farmland scale? China Popul Resour Environ. (2016) 26:97–104. doi: 10.3969/j.issn.1002-2104.2016.08.014

[51] Enahoro D Mason-D'Croz D Mul M Rich KM Robinson TP Thornton P. Supporting sustainable expansion of livestock production in South Asia and sub-Saharan Africa: scenario analysis of investment options. Glob Food Secur Agric Policy Econ Environ. (2019) 20:114–21. doi: 10.1016/j.gfs.2019.01.001

[52] Peng J Wu H Song J Li M. Impact of agricultural mechanization level on farmers' cropping index in Hubei Province. Chin J Eco-Agric. (2019) 27:380–90.

[53] Hawkes C Ruel MT From agriculture to nutrition: pathways, synergies and outcomes. Washington, DC: World Bank. (2008).

[54] Linderhof V Janssen V Achterbosch T. Does agricultural commercialization affect food security: the case of crop-producing households in the regions of post-reform Vietnam? Sustain For. (2019) 11:1263. doi: 10.3390/su11051263

[55] Matita M Chirwa EW Johnston D Mazalale J Smith R Walls H. Does household participation in food markets increase dietary diversity? Evidence from rural Malawi. Glob Food Secur Agric Policy Econ Environ. (2021) 28:100486. doi: 10.1016/j.gfs.2020.100486, PMID: 40226602

[56] Yang T Chandio AA Zhang A Liu Y. Do farm subsidies effectively increase grain production? Evidence from major grain-producing regions of China. Food Secur. (2023) 12, 12:1435. doi: 10.3390/foods12071435, PMID: 37048255 PMC10093900

[57] Ghose B. Food security and food self-sufficiency in China: from past to 2050. Food Energy Secur. (2014) 3:86–95. doi: 10.1002/fes3.48

[58] Kumar N Harris J Rawat R. If they grow it, will they eat and grow? Evidence from Zambia on agricultural diversity and Child undernutrition. J Dev Stud. (2015) 51:1060–77. doi: 10.1080/00220388.2015.1018901

[59] Zhong T Si Z Crush J Scott S Huang X. Achieving urban food security through a hybrid public-private food provisioning system: the case of Nanjing, China. Food Secur. (2019) 11:1071–86. doi: 10.1007/s12571-019-00961-8

[60] Shamdasani Y. Rural road infrastructure & agricultural production: evidence from India. J Dev Econ. (2021) 152:102686. doi: 10.1016/j.jdeveco.2021.102686

[61] Usman MA Callo-Concha D. Does market access improve dietary diversity and food security? Evidence from southwestern Ethiopian smallholder coffee producers. Agric Food Econ. (2021) 9:18. doi: 10.1186/s40100-021-00190-8

[62] Chen F Sun Z Zhao Y. The effects of social capital and family income on farmers’ participation in rural public goods provision. J Rural Stud. (2024) 109:103332. doi: 10.1016/j.jrurstud.2024.103332

[63] Aweke CS Lahiff E Hassen JY. The contribution of agriculture to household dietary diversity: evidence from smallholders in east Hararghe, Ethiopia. Food Secur. (2020) 12:625–36. doi: 10.1007/s12571-020-01027-w

[64] Habtemariam LT Gornott C Hoffmann H Sieber S. Farm production diversity and household dietary diversity: panel data evidence from rural households in Tanzania. Front Sustain Food Syst. (2021) 5:612341. doi: 10.3389/fsufs.2021.612341

[65] Kissoly LD Karki SK Grote U. Diversity in farm production and household diets: comparing evidence from smallholders in Kenya and Tanzania. Front Sustain Food Syst. (2020) 4:77. doi: 10.3389/fsufs.2020.00077

[66] Bennett MK. International contrasts in food consumption, Geogr. Rev. (1941) 31:365.

[67] Zhang X Zhang W. Is the development of grain-saving animal husbandry definitely beneficial to food security?—Empirical Analysis Based on Provincial panel Data. Ecological Economy, (2015) 31:113–116.

