# Supplementary material for: The impact of agricultural production diversity on farmer household dietary diversity: a case study of Nanjing City
Source: Front Nutr. 2025 Apr 30;12:1493371. doi: 10.3389/fnut.2025.1493371 (PMC12079581; doi:10.3389/fnut.2025.1493371)
Supplement: Supplementary file 1 [file Table_1.docx]

Supplementary Material

# Supplementary Data

**Survey on Agricultural Production Diversity**

**Household Head Characteristics**

1. Gender:

○ Male
○ Female

1. Year of Birth: ________ (e.g., 1970)
2. Ethnicity:

○ Han
○ Ethnic Minority

1. Educational Attainment:

○ Illiterate
○ Primary School
○ Junior High School
○ High School
○ Technical Secondary School
○ Associate Degree
○ Bachelor's Degree
○ Graduate Degree or higher

1. Marital Status:

○ Unmarried
○ Married
○ Divorced
○ Widowed

1. Health Status:

○ Dependent (unable to care for oneself)
○ Independent (able to care for oneself but lacks working capacity)
○ Capable of work but with health issues
○ Healthy

**Household Characteristics**

1. Total household members: ________, number of labor force: ________, number engaged in agriculture: ________, of which ________ also have non-agricultural occupations.
2. Household Structure:

○ Female-headed household without a husband or male partner (but with relatives, children, or friends)
○ Male-headed household without a wife or female partner (but with relatives, children, or friends)
○ Nuclear family (husband and wife, with or without children)
○ Extended family (husband, wife, children, and other relatives)
○ Other: _________________

1. Do you live with a pregnant woman or an infant (0-3 years old)?

○ I am pregnant, or I live with a pregnant woman
○ I live with an infant
○ Both
○ Neither

1. Is there a village official in your household?

○ Yes
○ No

1. Is there a member of the Communist Party in your household?

○ Yes
○ No

1. Has anyone in your household obtained a new-type professional farmer certificate?

○ Yes
○ No

1. Is your household a demonstration household for agricultural science and technology?

○ Yes
○ No

1. Primary occupation of the household:

○ Pure agriculture (agricultural income ≥ 90% of total household income)
○ Agriculture with part-time non-agricultural work (50% ≤ agricultural income < 90%)
○ Primarily non-agricultural with part-time agriculture (10% ≤ agricultural income < 50%)
○ Purely non-agricultural (agricultural income < 10%)

1. Investment in agricultural productive fixed assets (total household agricultural mechanization investment): ________ yuan
2. Total annual household income: ________ ten thousand yuan; annual agricultural income: ________ ten thousand yuan
3. Proportion of various income sources: ________% from crop cultivation, ________% from aquaculture, ________% from animal husbandry, ________% from forestry
4. Is your household income stable?

○ Stable
○ Fairly stable
○ Average
○ Unstable
○ Very unstable

1. Does your household have savings?

□ Current savings
□ Fixed-term savings
□ None

1. Total household expenditure in the past month: ________ yuan; food purchases: ________ yuan

**Agricultural Business Characteristics**

1. Which type of agricultural business entity best describes your household?

○ Smallholder (family-operated, total land area less than 30 mu)
○ Specialized large-scale household (family-operated, total land area over 30 mu, without business registration)
○ Family farm (family-operated, total land area over 30 mu, with business registration)
○ Leading enterprise in agricultural industrialization

1. Has your household registered as a business?

○ Yes
○ No

1. Are you a member of a farmer cooperative?

○ Yes
○ No

1. If yes, what subsidies or benefits has your household received?

□ Improved seed subsidy
□ Land transfer subsidy
□ Tax exemption
□ Water and electricity discounts
□ Other: _________________
□ No benefits received

1. What types of agricultural business entities do you cooperate with?

□ Smallholders (family-operated, less than 30 mu)
□ Specialized large-scale households (family-operated, over 30 mu, unregistered)
□ Family farms (family-operated, over 30 mu, registered)
□ Leading enterprises in agricultural industrialization
□ Farmer cooperatives
□ Village collective
□ None

1. Total number of cultivated plots: ________, total cultivated land area: ________ mu; area for cash crops (vegetables, fruits, tobacco, etc.): ________ mu; annual input in cultivated land: ________ yuan, output: ________ yuan
2. Total area of orchard: ________ mu, grassland: ________ mu, forestland: ________ mu, aquaculture area: ________ mu
3. Overall quality level of your cultivated land:

○ Excellent
○ Good
○ Average
○ Poor
○ Very Poor

1. Has the quality of your cultivated land changed since 2015?

○ Improved
○ Declined
○ No significant change

1. Main terrain of your cultivated land:

○ Primarily flat
○ Primarily sloped
○ Equal mix of flat and sloped

1. Degree of land fragmentation:

○ Consolidated
○ Fairly consolidated
○ Average
○ Fairly fragmented
○ Fragmented

1. Suitability of cultivated land for mechanization:

○ Fully suitable
○ Mostly suitable
○ About half suitable
○ Mostly unsuitable
○ Completely unsuitable

1. Main usage of agricultural products:

○ More than half for self-consumption
○ More than half for sale
○ Equally for self-consumption and sale

1. Crops cultivated over the past year and total area:

□ Grains, total area: _________________
□ Legumes, total area: _________________
□ Tubers, total area: _________________
□ Oilseeds, total area: _________________
□ Sugar crops, total area: _________________
□ Cotton, total area: _________________
□ Hemp, total area: _________________
□ Tobacco, total area: _________________
□ Medicinal plants, total area: _________________
□ Vegetables, total area: _________________
□ Tea, total area: _________________
□ Edible fungi, total area: _________________
□ Fruits, total area: _________________
□ Spices, total area: _________________
□ Ornamental plants, total area: _________________
□ Other: _________________
□ No crops cultivated

35. Which crops cultivated in your household are sold for income generation? What percentage of the total yield is sold for income?

□ Cereals, ___% of total yield sold for income

□ Legumes, ___% of total yield sold for income

□ Tubers, ___% of total yield sold for income

□ Oil crops, ___% of total yield sold for income

□ Sugar crops, ___% of total yield sold for income

□ Cotton, ___% of total yield sold for income

□ Hemp, ___% of total yield sold for income

□ Tobacco leaves, ___% of total yield sold for income

□ Medicinal herbs, ___% of total yield sold for income

□ Vegetables, ___% of total yield sold for income

□ Tea, ___% of total yield sold for income

□ Edible fungi, ___% of total yield sold for income

□ Melons and fruits, ___% of total yield sold for income

□ Spices, ___% of total yield sold for income

□ Flowers and potted plants, ___% of total yield sold for income

□ Other ___, ___% of total yield sold for income

36. Which aquatic products has your household raised in the past year? Total farming area (in mu)?

□ Fish, total farming area ______________

□ Crustaceans, total farming area ______________

□ Shellfish, total farming area ______________

□ Algae, total farming area ______________

□ Other _____, total farming area ______________

□ No aquatic products farmed

37. Which aquatic products raised by your household are sold for income? What percentage of the total yield is sold for income?

□ Fish, ___% of total yield sold for income

□ Crustaceans, ___% of total yield sold for income

□ Shellfish, ___% of total yield sold for income

□ Algae, ___% of total yield sold for income

□ Other ___, ___% of total yield sold for income

38. What livestock products has your household raised in the past year? Quantity raised (heads/animals)?

□ Cattle, quantity raised ______________

□ Donkeys, quantity raised ______________

□ Horses, quantity raised ______________

□ Mules, quantity raised ______________

□ Pigs, quantity raised ______________

□ Sheep, quantity raised ______________

□ Poultry, quantity raised ______________

□ Rabbits, quantity raised ______________

□ Bees, quantity raised ______________

□ Silkworms, quantity raised ______________

□ Other ___, quantity raised ______________

□ No livestock products raised

39. Which livestock products from your household are sold for income? Quantity sold (heads/animals)?

□ Cattle, ___ heads sold

□ Donkeys, ___ heads sold

□ Horses, ___ heads sold

□ Mules, ___ heads sold

□ Pigs, ___ heads sold

□ Sheep, ___ heads sold

□ Poultry, ___ heads sold

□ Rabbits, ___ heads sold

□ Bees, ___ heads sold

□ Silkworms, ___ heads sold

□ Other ___, ___ heads sold

40. What tree species are currently planted by your household? Plantation area (in mu)?

□ Timber forests, plantation area ______________

□ Economic forests, plantation area ______________

□ Shelter forests, plantation area ______________

□ Fuelwood forests, plantation area ______________

□ Special-purpose forests, plantation area ______________

□ Other _____, plantation area ______________

□ No trees planted

41. Which tree species provide income for your household?

□ Timber forests

□ Economic forests

□ Shelter forests

□ Fuelwood forests

□ Special-purpose forests

□ Other _____

42. Development status of other business activities beyond agricultural production?

□ Agricultural product processing

□ Agro-tourism (e.g., fruit-picking, eco-tourism)

□ Educational bases

□ Research land

□ Registered brand

□ Other _________________

□ None

43. Which agricultural product certifications has your household received?

□ Pollution-free agricultural product certification

□ Green food certification

□ Organic agricultural product certification

□ Geographical indication agricultural product certification

□ Good Agricultural Practices (GAP) certification

□ Other _________________

□ No agricultural product certifications

44. Household expenditure on agricultural production inputs (e.g., seeds, fertilizers, pesticides, aquaculture fry, livestock young stock, equipment, etc.) ________ CNY/year; total annual agricultural product sales revenue ________ CNY/year

45. Level of awareness regarding agricultural production-related policies?

○ Very knowledgeable

○ Quite knowledgeable

○ Average

○ Not very knowledgeable

○ Not at all knowledgeable

46. Primary sources of agricultural production and operation knowledge?

□ Personal experience and oral transmission from acquaintances

□ Reading books, newspapers, magazines, pamphlets, and field bulletin boards

□ Attending training, meetings, or university courses

□ Outreach by agricultural experts/technicians (including on-site guidance or demonstrations)

□ Self-study via internet

47. Has your household received any government subsidies for agricultural production?

□ Direct grain subsidies

□ Comprehensive agricultural input subsidy

□ Standardized pig farming facilities construction

□ Agricultural insurance subsidy

□ Minimum grain purchase policy

□ Reforestation subsidy

□ Grassland ecological protection subsidy

□ Special support (e.g., agricultural development loans with interest subsidies, industrialized operations, land management, support for "vegetable basket" products)

□ Other _________________

□ No agricultural subsidies received

48. Attitude towards agricultural risk?

○ Pursue income regardless of risk

○ Cautiously consider, avoid impulsive investment

○ Avoid any activity with risk

49. Does your household have agricultural insurance?

○ Yes

○ No

50. Have you or any family members attended agricultural technical training?

○ Attended ___ times/year

○ Never attended

51. Total duration engaged in agricultural production and operations ________ years, duration in Nanjing ________ years

**Market Participation**

52. What are the sales channels for agricultural products from your household?

□ Street vending

□ Agritourism (e.g., farm-based restaurants and experiences)

□ Sales to visiting merchants or delivery to wholesale markets

□ Sales to familiar supermarkets, processing plants, schools, companies, restaurants, etc. (without a contract)

□ Contracted sales with supermarkets, distribution companies, markets, or buyers

□ Online sales, e.g., through e-commerce platforms like Taobao or WeChat groups

□ Other _________________

53. Frequency of household food purchases (both online and offline) in the past week?

○ Never

○ Once per week

○ 2-3 times per week

○ 4-6 times per week

○ 7 or more times per week

54. Types of food purchased by your household in the past week?

□ Cereals (e.g., rice, noodles, rice noodles, bread, biscuits, or other foods made from rice, flour, sorghum, millet)

□ Tubers (e.g., potatoes, sweet potatoes, purple yams, taro, or other tuber foods and products)

□ Vegetables

□ Fruits

□ Meat (e.g., beef, pork, chicken, mutton, rabbit, duck, or other poultry and animal organs)

□ Eggs (e.g., chicken eggs, etc.)

□ Aquatic products (e.g., fish, dried fish, shellfish, and seafood)

□ Legumes (e.g., soybeans, peas, lentils, green beans, or bean products such as tofu, dried bean curd, bean curd skin)

□ Dairy products (e.g., cheese, yogurt, milk, or other dairy products)

□ Fats and oils (e.g., cooking oil, butter, nuts, etc.)

□ Sugar or honey

□ Spices (e.g., star anise, bay leaves, Sichuan pepper, cinnamon), coffee, and tea

55. Distance from your household to the nearest market (in kilometers)

*(Market includes large agricultural markets, small vegetable markets, supermarkets, and similar marketplaces where food can be purchased)*

56. The most commonly used mode of transportation and travel time to reach the market from your household?

○ Walking, ________ minutes

○ Bicycle, ________ minutes

○ Electric bike, ________ minutes

○ Motorcycle, ________ minutes

○ Public bus, ________ minutes

○ Car, ________ minutes

○ Other ________, travel time ________ minutes

57. How would you rate the prices of food at the market?

○ Very cheap

○ Relatively cheap

○ Average

○ Relatively expensive

○ Very expensive

○ No information

58. What types of food did you consume in the past 24 hours?

□ Cereals (e.g., rice, noodles, rice noodles, bread, biscuits, or other foods made from rice, flour, sorghum, millet)

□ Tubers (e.g., potatoes, sweet potatoes, purple yams, taro, or other tuber foods and products)

□ Vegetables

□ Fruits

□ Meat (e.g., beef, pork, chicken, mutton, rabbit, duck, or other poultry and animal organs)

□ Eggs (e.g., chicken eggs, etc.)

□ Aquatic products (e.g., fish, dried fish, shellfish, and seafood)

□ Legumes (e.g., soybeans, peas, lentils, green beans, or bean products such as tofu, dried bean curd, bean curd skin)

□ Dairy products (e.g., cheese, yogurt, milk, or other dairy products)

□ Fats and oils (e.g., cooking oil, butter, nuts, etc.)

□ Sugar or honey

□ Spices (e.g., star anise, bay leaves, Sichuan pepper, cinnamon), coffee, and tea

59. Name of the street (town) and village committee (community committee) where you reside

_____ Street (Town), _____ Village Committee (Community Committee)

60. After completing the questionnaire, the investigator should submit the geographical location on-site:
